# Upright Radiation Therapy—A Historical Reflection and Opportunities for Future Applications

**DOI:** 10.3389/fonc.2020.00213

**Published:** 2020-02-25

**Authors:** Sulman Rahim, James Korte, Nicholas Hardcastle, Sarah Hegarty, Tomas Kron, Sarah Everitt

**Affiliations:** ^1^Department of Radiation Therapy, Peter MacCallum Cancer Centre, Melbourne, VIC, Australia; ^2^Department of Biomedical Engineering, School of Engineering, University of Melbourne, Melbourne, VIC, Australia; ^3^Department of Physical Sciences, Peter MacCallum Cancer Centre, Melbourne, VIC, Australia; ^4^Centre for Medical Radiation Physics, University of Wollongong, Wollongong, NSW, Australia; ^5^Department of Physics, RMIT University, Melbourne, VIC, Australia; ^6^Peter MacCallum Cancer Centre, Melbourne, VIC, Australia; ^7^Sir Peter MacCallum Department of Oncology, Faculty of Medicine, Dentistry and Health Sciences, University of Melbourne, Parkville, VIC, Australia

**Keywords:** radiation therapy, upright, CBCT (cone beam computed tomography), dosimetry, unconventional, patient positioning, treatment techniques

## Abstract

Since the early days of megavoltage Radiation Therapy (RT), the potential of delivering treatment to a sub group of patients in an upright position has been recognized. Compared to lying horizontally, treating patients in an upright position offers potential benefits in terms of patient comfort especially for patients experiencing dyspnoea and saliva accumulation when lying down. Dosimetric benefits can also be gained from changes in the volume and location of lungs and heart in an upright position, which are potentially advantageous for clinical situations including Hodgkin's disease, lung and breast malignancies. Since the 1950's, upright stabilization mechanisms have ranged from standalone chair based apparatus to couch-top attachments with increasingly customizable solutions. The introduction of Computed-Tomography (CT) based three-dimensional (3D) dosimetry in the 1980's−90's necessitated image acquisition in a horizontal position (supine or prone), significantly reducing options for alternative patient positioning and upright techniques. Despite this, upright techniques have still been utilized where clinically indicated for palliative and novel approaches often involving non-standard treatment scenarios. More recently, a small number of centers have reported on specialized equipment capable of acquiring planning data with the patient in a vertical position. The possibility of acquiring planning quality Cone Beam CT (CBCT) on linear accelerators has recently reinvigorated the potential to deliver highly accurate and targeted treatments to patients in an upright position. This paper reflects on the historical applications of upright RT and explores new possibilities for this technology in modern RT departments.

## Introduction

As one of the four major pillars in cancer management, Radiation Therapy (RT) is an important treatment modality providing curative and palliative intent management for a wide range of tumors (1). Establishing a suitable treatment position to enable the delivery of a therapeutic radiation dose while respecting tolerances of healthy surrounding organs is a fundamental step in the planning process of RT (2). Patient positioning and stabilization has significantly evolved since the early use of this modality, facilitating tighter margins, and higher doses (2).

Modern day radiotherapy is primarily administered with the patient lying in a supine or prone horizontal position. This is necessitated by the reliance on consistency with diagnostic imaging studies, such as Computed Tomography (CT), that are acquired in a horizontal position and are required for radiation dose calculations. However, some patients prescribed radiation therapy may benefit from non-conventional treatment positioning, such as upright positioning. Compared to horizontal orientations, gravitational changes afforded with upright positioning may prove beneficial for the irradiation of certain tumor volumes and critical organs at risk (OAR). Further, patients experiencing dyspnoea or excessive saliva accumulation when lying down may prefer a more comfortable stance.

The aim of this paper is to evaluate immobilization systems, clinical indications, image acquisition, and planning methods for upright radiation treatments over time. Opportunities for upright developments are also identified for future applications.

## Search Strategy

A comprehensive search strategy through the indexed databases (Medline & Embase) was performed with no date restriction. The search query used was as follows:

((upright or seat or seated or sitting or standing or chair) adj3 (radiotherapy or radiation or irradiation or simulation or planning or proton or particle or imaging or immobiliation or position*)).mp.

OR

(cancer* or neoplasm* or tumor* or carcinoma* or malignanc* or oncolog* or leukemia* or lymphoma* or metasta*).mp.

The question mark and asterisk indicate “wildcard” characters that allow different entries in the search. Relevant papers, including relevant cited references in these papers, published in English were included.

## Immobilization Systems

Standalone upright immobilization solutions have historically been used with varying degrees of complexity. One of the earliest reports of upright RT was made more than 60 years ago by Morrison et al. (3), who reported the use of back, arms, and head support along with footrest attachments to the treatment bed to form an upright stabilization apparatus (3). Stabilization solutions from this era aligned with the simple treatment techniques of the time and still managed to deliver therapeutic doses to large treatment areas e.g., for Hodgkin's Lymphoma.

**Figure 1:**
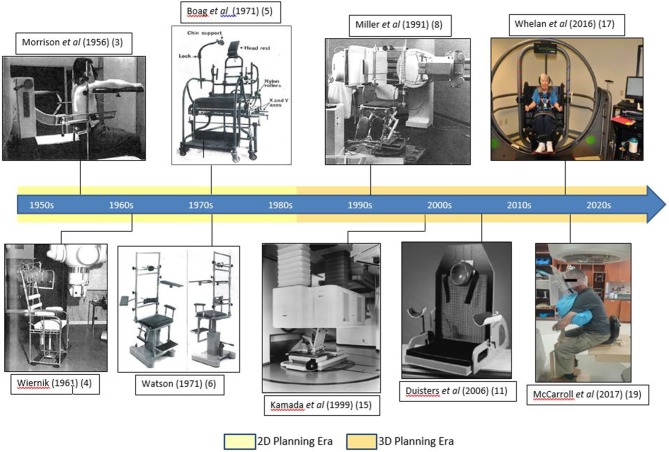
Upright patient setup mechanisms as shown in the literature.

Chair designs gradually became more specialized for specific treatment sites, including head and neck as described by Wiernik and Boag et al. and for thorax as described by Watson et al. (4–6). Figure 1 shows the evolution of upright chair design for treatment delivery using a linear accelerator.

Seated positioning has also been employed as a necessity in cases of fixed horizontal beam using protons, where the large physical size of the beam producing mechanism made it impossible to rotate the gantry around the patient. Verhey et al. described patient stabilization in front of the gantry using facemasks and, in some cases, a rigid bite to maintain treatment position for head and neck tumors with radiographs used for target area verification (7). This stabilization approach reported mean intra-fraction motion to be as little as 0.8 mm (7).

Miller et al. (8) presented an isocentric chair that overcame some of the limitations of the previous models enabling precise isocentric horizontal and vertical localization and rotation about a vertical axis. This mobile chair allowed for anterior/posterior beam entry and also oblique fields at normal or extended Source-Axis Distance (SAD) (8). Better OAR sparing was reported in the case of bulky mediastinal disease treated using a mantle technique with reduced lateral tumor spread in the seated position established by comparing simulator films in the two positions (8).

Marcus et al. reported the use of a standard upright chair for administering mantle irradiation (9). The upright position reduced the width of the treatment area and heart, thereby allowing a greater volume of lung to be shielded. Ultimately, the upright technique enabled bulky mediastinal Hodgkin's disease to be safely treated with higher doses, with no increase in relapse or toxicity. The limitation of the upright position was reduced positional reproducibility, with over 70% of the pre-treatment radiographs requiring adjustment of the shielding blocks by over 5 mm limit relative to over 90% of patients treated in supine position being within that limit (9). Soon after, Klein et al. reported the use of a commercial chair solution for thorax irradiation, reporting increased accuracy and as much as 60% additional lung sparing when comparing upright radiographs to supine and prone position (10).

More recently, Duister's et al. compared supine to seated positions for palliative chest irradiation by simulating 10 patients in seated, supine and then seated again using a commercially available chair (MT-2000 Treatment chair, Med-Tec Inc.) (Figure 1) (11). Anterior-posterior (AP) treatment fields and lungs were marked on the simulated films. The seated position was reported to be reproducible with significantly higher total lung volume, thereby reducing the percentage of irradiated lung and all patients indicated a preference for upright positioning (11).

## Clinical Indications

**Figure 2:**
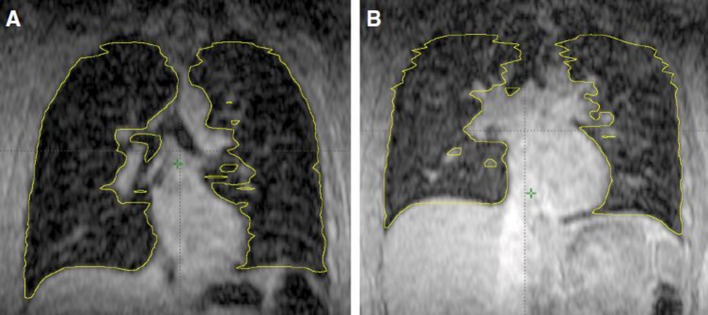
Coronal view of lung volume at the end of exhalation in seated position left **(A)** and in supine position right **(B)** as shown by Yang et al. (12).

Upright treatment positioning is a novel treatment option when traditional supine or prone treatment options are inferior for clinical reasons. These include palliative intent treatments, where patients find it difficult to lie down e.g., with superior vena cava obstruction or pleural effusion. Upright positioning also offers benefits for lung cancer patients considered for treatment with curative intent, as shown by Yang et al. who compared lung volumes of five healthy volunteers in the upright and supine position using cine MR images (12). The average end-of-exhale lung volume was 27% larger in the seated position and offered reduced motion within the lung for all patients (Figure 2). Although gains in lung volume were greater with Deep Inspiration Breath Hold (DIBH), it provides a useful alternative position to decrease the mean lung dose and potentially enhance patient comfort and reproducibility (12).

Shah et al. reported a case for a patient unable to lie down due to phrenic nerve damage following post radiation surgical intervention for thymic cancer (13). A modified GE CT 8800 model (General Electric, Milwaukee, WI) CT scanner that descended vertically over the standing patient acquired axial images for dose calculation. The patient was positioned upright for a fractionated re-irradiation using photons and electrons with custom stabilization equipment made for treatment setup reproducibility (13).

Another novel application was reported by Mohiuddin et al., for a 34 year old morbidly obese female patient prescribed post lumpectomy whole breast treatment who was unable to receive radiotherapy in conventional position due to treatment bed weight limitations (14). The patient was positioned next to the edge of the treatment couch with the treatment breast placed over an attached carbon-fiber board and a mouldable cushion supporting and defining the breast contour (14). A thermoplastic cast was used over the breast to localize and help define the breast contours which were palpated and marked with opaque wires (14). The empty breast mold was CT scanned and a tissue equivalent density was assigned to treatment volume representing the actual breast for dose calculation (14). This approach successfully facilitated breast conservation therapy and the patient received 50 Gy in 25 fractions without any interruptions with mild grade two skin toxicity (14).

## Upright Image Acquisition and Planning Methods

Although chest irradiation in an upright position has been effectively utilized in the past and its advantages such as increased lung volume and patient comfort acknowledged; the inability of acquiring an upright CT for dose planning remains a major barrier preventing the adoption of upright RT in modern day scenarios (3, 4, 6, 8).

Only a small number of centers have reported their experience with upright CT acquisition for RT planning purposes. Kamada et al. reported seated stabilization for treatment planning and verification designed for a fixed horizontal heavy-ion beam line using carbon ions (Figure 1) (15). The 3D dose calculation was performed on CT images acquired by moving a CT scanner vertically over the patient seated in the treatment position (15).

Rotating the patient instead of the gantry for image acquisition and dose modulation was suggested by Eslick et al. in their low cost compact system named Nano-X (16). This fixed beam system with fewer shielding requirements potentially offers an attractive alternative solution for both rural and crowded metropolitan RT departments. Challenges of this system may include organ deformation with patient rotation and patient comfort (16).

Whelan et al. used a device usually employed to treat balance disorder to slowly rotate a sample of 10 cancer survivors through a single 360° rotation while sitting up (Figure 1) and lying down (17). Their preliminary results showed good tolerance for slow rotation for treatment delivery with no reported anxiety and motion sickness when sitting up with only one patient unable to complete the full arc rotation lying down (17).

Most recently, attempts to utilize existing technologies for image acquisition have been reported. Fave et al. reported a method of acquiring CBCTs in an upright position for dose calculation using a TrueBeam linac (Varian, Palo Alto, CA) (18). Upright CBCT was acquired by rotating the treatment couch 180° and keeping the gantry, kV source and detector stationary (18). The extended Field Of View (FOV) required to capture the entire anatomy of the anthropomorphic phantom was achieved by taking two scans with detector offsets for each acquisition and stitching them together (18). Hounsfield Unit (HU) mapping from a simulation CT was used to account for inaccuracies introduced by this method (18).

McCarroll et al. recruited five patients with an indication for head and neck RT and simulated an upright treatment using the chair apparatus (Figure 1) with reproducibility tested for both intra- and inter-fraction scenarios using KV images (19). The images were assessed in different subregions of the head and neck anatomy with average intra-fraction displacement found to be <2 mm and <3 mm for inter-fraction displacement for the entire patient sample (19). This set-up was concluded to be feasible for CBCT imaging with its possible use for dose calculation and treatment (19).

## Discussion

Despite early endeavors to administer upright RT, the evolution of routine CT based dose simulation resulted in this approach only being contemplated as a “last resort” option for end of life palliation or, conversely, for highly complex treatments where specialized CT apparatus existed to acquire planning data in the upright position. However, upright positioning could provide a useful alternative for situations demanding unconventional approaches due to clinical, equipment driven or patient preference reasons. With the evolution of CBCT and the promise of its use for dosimetry calculation, the conversation regarding the upright treatment position for radiation therapy is re-emerging. Our team is currently studying the technical and clinical feasibility of integrating CBCT acquisition into the RT dosimetry calculation workflow. While experiments are still at a developmental phase, outcomes of work to date suggest that clinically acceptable image quality can be yielded on conventional linac CBCT.

Novel treatment delivery ideas such as moving the patient instead of the gantry to acquire planning images as well as treatment delivery open a multitude of treatment scenarios where treatment positions other than conventional supine or prone are now being explored (16–19). Traditional horizontal stabilization solutions have undergone continuous research and development and refined iterations over many decades. Accurate and reproducible solutions, including robust radio-lucent stabilization systems, will also be required for treatments in this unconventional scenario. Upright positioning could also be employed in fixed gantry solutions using particle therapy or lower cost LINAC offering potential health economic benefits. Such a system also carries the potential to be housed in a mobile device e.g., a truck or a ship, making radiotherapy more accessible to remote and regional locations.

Learnings from earlier upright stabilization systems along with more recent studies showing anatomical changes associated with upright positioning provide enough evidence to investigate this alternative approach in a modern day radiation therapy context. Furthermore, other anatomical sites such as breast could also exploit the anatomical changes of increased lung volume and isolation of treatment area from OARs in an upright position. Once the hurdle of acquiring planning quality images is overcome, upright positioning could provide a certain patient demographic a more comfortable and potentially clinically equivalent if not superior option for radiation therapy.

## Author Contributions

All authors listed have made a substantial, direct and intellectual contribution to the work, and approved it for publication.

### Conflict of Interest

The authors declare that the research was conducted in the absence of any commercial or financial relationships that could be construed as a potential conflict of interest.
